# Self-assembly and photoinduced fabrication of conductive nanographene wires on boron nitride

**DOI:** 10.1038/s41467-021-27600-1

**Published:** 2022-01-21

**Authors:** Xiaoxi Zhang, Fabian Gärisch, Zongping Chen, Yunbin Hu, Zishu Wang, Yan Wang, Liming Xie, Jianing Chen, Juan Li, Johannes V. Barth, Akimitsu Narita, Emil List-Kratochvil, Klaus Müllen, Carlos-Andres Palma

**Affiliations:** 1 Beijing National Laboratory for Condensed Matter Physics, Institute of Physics, Chinese Academy of Sciences, Beijing, 100190 PR China; 2 School of Physical Sciences, University of Chinese Academy of Sciences, Beijing, 100049 PR China; 3 Department of Physics, Department of Chemistry & IRIS Adlershof - Humboldt-Universität zu Berlin, 12489 Berlin, Germany; 4 Max Planck Institute for Polymer Research, Ackermannweg 10, 55128 Mainz, Germany; 5 State Key Laboratory of Silicon Materials, Zhejiang University, Hangzhou, 310027 PR China; 6 College of Chemistry and Chemical Engineering, Central South University, Changsha, 410083 PR China; 7 School of Physics, Beijing Institute of Technology, Beijing, 100081 PR China; 8 National Center for Nanoscience and Technology, Beijing, 100190 PR China; 9 Advanced Research Institute for Multidisciplinary Science, Beijing Institute of Technology, Beijing, 100081 PR China; 10 Physik-Department E20, Technische Universität München, D-85748 Garching, Germany; 11 Helmholtz-Zentrum Berlin für Materialien und Energie GmbH, 14109 Berlin, Germany; 12 Department of Physics & IRIS Adlershof - Humboldt-Universität zu Berlin, 12489 Berlin, Germany

**Keywords:** Molecular self-assembly, Nanoscale devices

## Abstract

Manufacturing molecule-based functional elements directly at device interfaces is a frontier in bottom-up materials engineering. A longstanding challenge in the field is the covalent stabilization of pre-assembled molecular architectures to afford nanodevice components. Here, we employ the controlled supramolecular self-assembly of anthracene derivatives on a hexagonal boron nitride sheet, to generate nanographene wires through photo-crosslinking and thermal annealing. Specifically, we demonstrate µm-long nanowires with an average width of 200 nm, electrical conductivities of 10^6 ^S m^−1^ and breakdown current densities of 10^11 ^A m^−2^. Joint experiments and simulations reveal that hierarchical self-assembly promotes their formation and functional properties. Our approach demonstrates the feasibility of combined bottom-up supramolecular templating and top-down manufacturing protocols for graphene nanomaterials and interconnects, towards integrated carbon nanodevices.

## Abstract (web summary)

The bottom-up fabrication of structures with robust performance in the nm-to-μm scale usable for integrated carbon nanodevices is challenging. Here the authors report micrometer-long, highly conducting nanographene wires following self-assembly, photo-crosslinking and thermal annealing of anthracene derivatives on hexagonal boron nitride sheets.

## Introduction

Complex material manufacturing through self-assembly is currently approaching atomic precision^1,2^, which is instrumental for the advancement of modern carbon nanodevices. In parallel to bulk preparation, surface-templated fabrication offers unique advantages for interfacial molecular architecture self-assembly and synthesis, such as rational atomistic design aided by joint computational modeling and scanning probe microscopy investigations^3–9^. Of fundamental interest is to establish a link between on-surface self-assembly and synthesis, and the fabrication of bottom-up structures with robust performance in the nm-to-μm scale usable for integrated carbon nanodevices. In principle, interfacial molecular architectures can be leveraged to obtain robust, functional nanoscale components via photolithography or similar top-down patterning strategies^10^. Towards the fabrication of precision carbon interconnects, resistors, diodes, supercapacitors, and other electronic components^11^, it is favorable to crosslink supramolecular architectures into nanosheets or nanowires^12,13^, and control their processing by, e.g., ultra-fast Joule heating^14^ or thermal conversion^15,16^. However, atomic precision and functionality in the context of nanodevices remain largely unexplored in interfacial molecular architectures. Regarding atomic precision, a major challenge is to control the organization of the architecture to induce advantageous covalent reaction pathways^17–23^ using for example a variety of homolytic cleaving groups to induce crosslinking^10,20,21,24,25^. Concerning functionality, two major obstacles regarding applications are moderate device performances and processing conditions incompatible with nanofabrication. For modern nanomanufacturing, it is desirable to template supramolecular architectures in device configurations of choice, and react them in situ to reach the target technology, such as broadband and conductive (e.g., 10^7 ^S m^−1^)^26^ nanoscale interconnects together with state-of-the-art logic elements (e.g., transistor or quantum circuits). Overall, an important frontier of atomically-precise bottom-up science is to attempt to engineer nanodevices, by stimuli-induced crosslinking or thermal annealing of increasingly regular supramolecular architectures^18,25,27,28^ at device interfaces.

**Fig. 1:**
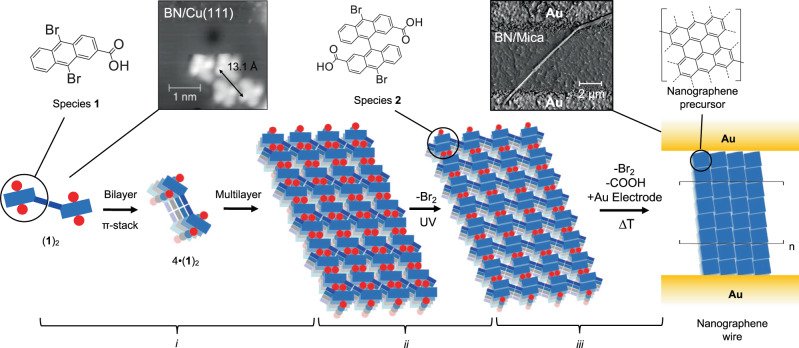
Hierarchical self-assembly, crosslinking and thermal conversion of supramolecular structures. Species **1** undergoes supramolecular H-bond dimerization, π-stacking and *vdW* layered growth into supramolecular nanowires (*i*). Species **2** can be formed by ultraviolet (UV) photo-crosslinking within the supramolecular architectures formed by species **1** (*ii*), and subsequently undergoes thermal dehydrogenation to nanographene precursors and thermal crosslinking into nanographene networks (*iii*). The so-formed nanographene wires are defined by the nanographene material retaining their supramolecular architecture wire-like scaffold. The inset in (*i*) shows the scanning tunneling microscopy (STM) data of four flat-on molecules on the BN/Cu(111) surface, forming two (**1**)_2_ supramolecular dimers, held together by an interrow hydrogen bond. The inset in (*iii*) shows the scanning electron microscopy (SEM) of the final nanographene wire between electrodes. STM parameters: *I*_*t*_ = 80 pA, *V*_*s*_ = 1.0 V. SEM parameters 2 kV, *I* = 10 μA.

Herein we adopt a surface-templated approach to fabricate and monitor supramolecular nanoarchitectures on hexagonal boron nitride (BN), and their conversion into highly conducting nanowires (10^6 ^S m^−1^, Fig. 1) between gold electrodes in situ. In an ultra-high vacuum (UHV) environment, we thermally sublimated precursor molecules, brominated anthracene carboxylic acid derivative **1** on BN, to guide hydrogen-bonding and hierarchical assembly of the molecules^29,30^. Joint molecular dynamics (MD), scanning tunneling microscopy (STM) and atomic force microscopy (AFM) studies reveal that the molecular units form a periodic pattern according to the expected π-stacking motif with *d* = 3.5 ± 0.2 Å at the BN/Cu(111) interface. Supramolecular architectures grow upward in the direction perpendicular to the surface of the BN substrate, and supramolecular nanowires evolve with a micrometer length, as characterized by scanning electron microscopy (SEM). After their formation, ultraviolet (UV) light is employed to induce debromination of supramolecular structures of **1** to form species **2**, monitored by matrix-assisted laser desorption ionization (MALDI) mass spectrometry (MS)^24^. Species **2** engages subsequently in dehydrogenative coupling, affording nanographene wires, whereby Raman spectroscopy indicates further dehydrogenative chemical conversions following 1273 K annealing, providing highly regular nanowires. Mean conductivities of (1.6 ± 2.0) × 10^6 ^S m^−1^ (19.7 ± 7.3 Ω per μm of a 200 nm diameter nanowire) and breakdown current densities of (1.6 ± 1.9) × 10^11 ^A m^−2^ further substantiate successful nanowire synthesis on insulators.

## Results & discussion

### Simulations and STM of interfacial self-assembly

**Fig. 2:**
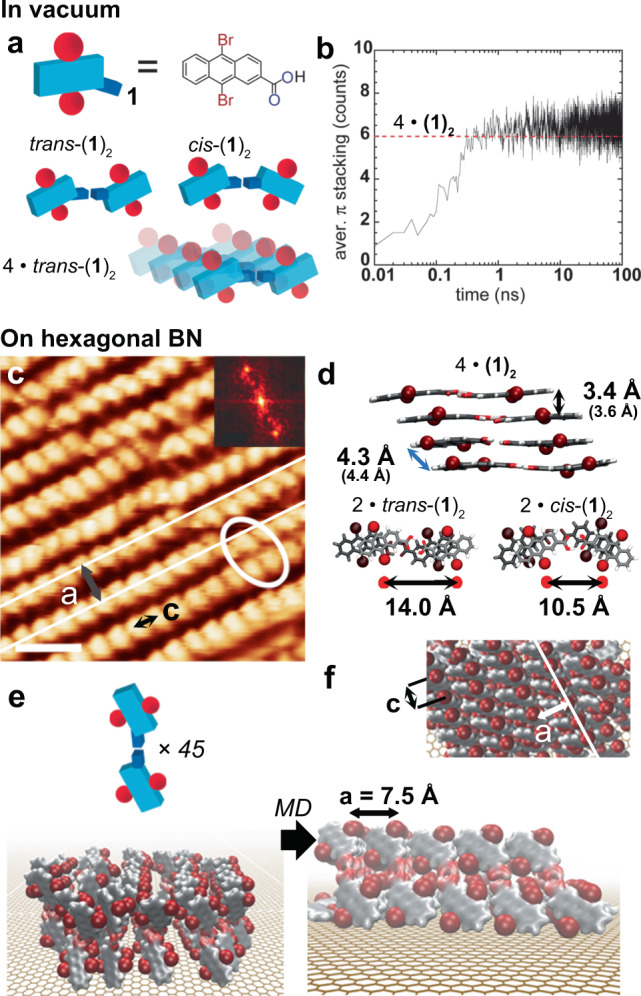
Simulations and scanning tunneling microscopy detail of the first-layer self-assembled stacks of (1)_2_ on boron nitride. **a** H-bond and ${{{{{\rm{\pi }}}}}}$-stacking of **1** may promote densely packed rows. **b** In vacuo molecular dynamic (MD) simulations of ten molecules of **1** in a 125 nm^3^ box show ultrafast propensity towards hydrogen bond dimerization into (**1**)_2_ and ${{{{{\rm{\pi }}}}}}$-stacking: After 1 ns the number of ${{{{{\rm{\pi }}}}}}$-stacks (6–8) indicate the formation of 4•(**1**)_2_ stacks. **c** Ultra-high vacuum scanning tunneling microscopy (UHV STM) data at ~20 K reveals supramolecular structures with periodicity c = (3.5 ± 0.2) Å on BN/Cu(111). Scale bar 1 nm. STM parameters *I*_*t*_ = 80 pA, *V*_*s*_ = 1.0 V. In the straight pattern the interrow distance is a = 6.4 Å. **d** Dispersion-corrected density functional tight binding (DFTB) optimization of a 4•(**1**)_2_ stack extracted from the MD simulations, containing two stacks of trans-(**1**)_2_ and two of cis-(**1**)_2_ with *d* = 3.4 Å. The numbers in parenthesis refer to calculations employing a Lennard-Jones potential. **e**, **f** Molecular dynamics of 45 upstanding edge-on species of (**1**)_2_ on BN reveals self-assembly in regular patterns with an interrow distance of a = 7.5 Å, c = 3.7 Å. Inset: Top-view of the assembly after 10 ns.

Brominated anthracene was successfully employed for on-surface synthesis of polyanthrenes and ultra-narrow graphene nanoribbons^31^. Nanographene formation involves three steps: debromination, free radical-radical coupling, and thermal dehydrogenation^32,33^. On metal surfaces, the success of the reaction relies on efficient diffusion and low steric hindrance between the radical intermediates^32,33^. On insulators, the debromination can be assisted by light, but to date yielded amorphous materials^24^. Notwithstanding this, ordered assemblies with closely packed bromines can promote radical-radical coupling^24,25^. Precursor **1**, 9,10-dibromo-anthracene-2-carboxylic acid, favors expression of supramolecular dimers via carboxylic acid H-bonds, (**1**)_2_, providing the constituents for robust ${{{{{\rm{\pi }}}}}}$-stacking complexes (Fig. 2a). Following this strategy, adjacent Br atoms can be abstracted by the stimulation with UV light, potentially promoting directional radical-radical coupling^24,25^. To understand the propensity of the precursors towards hierarchical assembly via H-bonding and subsequent ${{{{{\rm{\pi }}}}}}$-stacking, several CHARMM force field^34^ molecular dynamic (MD) simulations were performed for ten randomly distributed molecules in vacuo. After 1-ns simulations at temperatures between 370 K and 400 K, an average number of six ${{{{{\rm{\pi }}}}}}$-stacks indicates the formation of a 4•(**1**)_2_ supramolecular stacks (Fig. 2b). One representative 4•(**1**)_2_ stack extracted from the MD simulations and optimized by self-consistent charge, dispersion corrected, third-order density functional tight binding (SCC D-DFTB3) employing the DFTB + package^35^ is depicted in Fig. 2c. The cluster consists of two stacks of cis-(**1**)_2_ and two of trans-(**1**)_2_. The ${{{{{\rm{\pi }}}}}}$-stack distances amount to *d* = 3.4 Å for the Grimme implementation of dispersion correction^36^ (D3) and *d* = 3.6 Å for a Lennard Jones (LJ) implementation. These two methods usually correspond to lower and upper estimates, respectively, of common stacking distances^37,38^.

To study self-assembly and crosslinking under equivalent conditions, a monolayer of **1** (cf. Supplementary Synthesis, and Supplementary Figs. 1 and 2) was sublimated (30 min, 433 K) under UHV (10^−9^ mbar) on top of a model substrate for inert self-assembly^39^: An hexagonal BN monolayer on Cu(111)^40^. The sublimation temperatures employed are much below the decomposition temperature of **1** as measured by thermal gravimetric analysis (>677 K). Cryogenic STM data (Fig. 2d) reveals the supramolecular structure is characterized by rows with an interrow periodicity **a** and intrarow periodicity **c** = (3.5 ± 0.2) Å. The interrow distance is closer than previously reported anthracene carboxylic acid structures (~3.9 Å)^41^. For straight patterns, **a** amounts to 6.4 Å. To elucidate probable molecular arrangements, departing from a supramolecular dimer (**1**)_2_, we built a disordered cluster featuring 45, upstanding, trans-(**1**)_2_ dimers on BN (Fig. 2e). After a few ns of MD simulations at 295 K (Fig. 2f), the cluster self-assembled into a brick structure^42^ with **c** = 3.7 Å and interrow distances of **a** = 7.5 Å, in close agreement with experimental data. We assign the ~1 Å interrow discrepancy with experiment to the finite size of our cluster, since larger clusters or structures induce denser configurations. It is worth noting that lower deposition times result in flat-on molecular arrangements on the BN/Cu(111) substrate, as shown in the STM data inset in Fig. 1 and Supplementary Fig. 3. Polymorphic domains in large area STM surveys (Supplementary Fig. 4) reveal isolated rows, without neighbors or with odd-number of neighboring rows, which cannot account for in-plane H-bond formation (cf. Supplementary Fig. 3). Hence, the upstanding π-stacked (**1**)_2_ rows are likely held together by interrow *vdW* interactions only. The absence of interrow distances comparable to the H-bonded cis or trans interdimer distance of ~10–14 Å additionally substantiates this conclusion (cf. dimer length in Fig. 1 inset with Fig. 2c and Supplementary Figs. 3 and 4). This type of upstanding molecular orientation is possible for multilayers of acenes^43–45^ and benzothiophenes^46^ on graphene and BN, where interrow distances are close to 6 Å. These exhaustive STM and MD simulations identify three hierarchical levels of assembly: carboxylic acid dimer formation, intrarow π-stacking, and interrow *vdW* interactions.

### Simulations and characterization of supramolecular nanowires

**Fig. 3:**
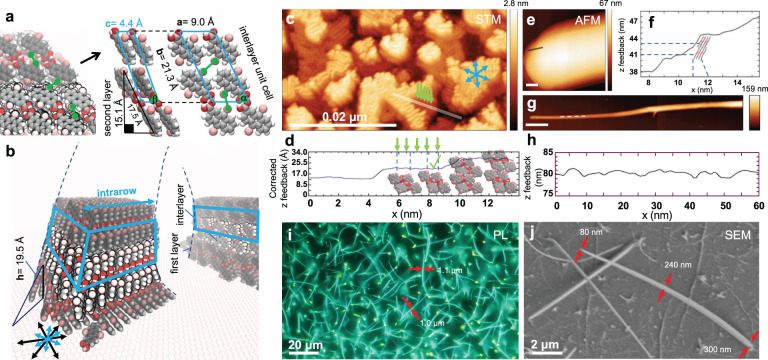
Simulations, analysis and ultraviolet irradiation of supramolecular nanowires. **a** A simulated interlayer (**1**)_2_–(**1**)_2_ unit cell on top of the first-layer and, **b** overview of ns-long molecular dynamics at 350 K depicting nanowire pillars on hexagon-boron nitride (BN). Molecules loosely adsorbed on the substrate have been removed for clarity. The cyan boxes depict the interlayer region supercells, and the intrarow direction (blue arrow) is shown perpendicular to the substrates’ high symmetry directions (black arrows). **c** Ultra-high vacuum scanning tunnel microscopy (UHV STM) data at ~20 K of islands of **1** after 30 min multilayer deposition on BN/Cu(111). The blue arrows depict the row directions. **d** The apparent height profile along two strata in **c**. Standing (**1**)_2_ dimers on the substrate are shown to scale. The rows are independent of each other and the average interrow separation (green arrows) is <1 nm, similar to the interrow parameter in Fig. 2. STM parameters *I*_*t*_ = 80 pA, *V*_*s*_ = 800 mV. **e**, **g** Atomic force microscopy (AFM) detail of tip of a nanowire after 60 min multilayer deposition on BN/mica. The scale bars in **e** and **g** are 7 nm and 50 nm, respectively. **f** The profile of **e** depicts eight stacked (**1**)_2_ shown to z-scale and ~2 nm height variations along the wire. **h** The profile of **g** depicts ~2 nm height variations along the wire. **i** Photoluminescence (PL) (*λ*_ex_ = 350 nm) microscopy showing supramolecular nanowires after 60 min multilayer deposition on BN/mica and corresponding diffraction-limited widths (red arrows). **j** Scanning electron microscopy of the structures after ultraviolet (UV) irradiation, cf. structures before irradiation Supplementary Fig. 7. SEM parameters 1 kV, *I* = 10 μA.

Microsecond-long simulations of 576 molecules at 350 K were employed to complementarily study (**1**)_2_ multilayered stacking perpendicular to the substrate surface, that is, in three dimensions (3D). The simulations reveal that brick-packed layers can grow in a layer-by-layer manner, stacking in 3D, with a triclinic unit cell extracted from the steepest descent optimization of the (**1**)_2_–(**1**)_2_ interlayer motif amounting to **a** = 9.0 Å, **b** = 21.3 Å, **c** = 4.4 Å with *α*, *β*, *γ* = 63°, 68°, 80°, respectively (Fig. 3a, b). Preparations employing higher deposition fluxes (30 min at 453 K, 10^−9^ mbar) at a BN/Cu(111) substrate temperature of 353–373 K evidence layered growth (Fig. 3c), whereby the STM data in Fig. 3c depicts molecular islands associated with stacked layers on top of each other. The line profile in Fig. 3d shows an apparent height of the first step of 21 Å. This height is close to the Br–substrate distance of the first layer, amounting to **h** = 19.5 Å (triangle, Fig. 3b) and is similar to the projection of the length of (**1**)_2_ in Fig. 3a to the substrate (15.1 Å), plus the adsorption height. Considering an adsorption height of 3.4 Å, the discrepancy (*Δh* = 19.5 Å – 18.5 Å) is assigned to a steeper first layer on the surface as compared to the unit cell. The interrow distances (blue arrows, Fig. 3b) and height of the first step are too far apart and high, for anything but two upstanding (**1**)_2_, in accordance with Fig. 2 data. However, the STM apparent height difference between the first and second layer (8 Å Fig. 3d, cf. Supplementary Fig. 5) is lower than the simulated bilayer height in Fig. 3b (15.1 Å). Because the rows of the second layer are very similar to those of the first layer, we assign the height discrepancy to electronic or vibronic tunneling effects. Note how the rows are oriented perpendicular to the substrate’s high symmetry directions, an effect which is reproduced in the simulations due to bromine and hydrogen preferring the hollow sites of the honeycomb lattice in molecular mechanics.

In further studies below, we employed longer deposition times aiming at slow, multilayered growth of supramolecular architectures. For these studies the substrate BN was transferred from copper to mica sheet. After 60 min deposition, supramolecular nanowires of **1** are further evidenced in AFM data (Fig. 3e–h). Height profiles are shown along the tentative growth directions, where step heights and lengths of ~3 nm can be resolved (Fig. 3e, f). Photoluminescence (PL) microscopy reveals that the supramolecular structures form ~20-µm-long nanowires on BN surface upon 60 min deposition, featuring apparent, diffraction-limited widths of ~1 µm (Fig. 3g, i). The PL identifies the supramolecular peak emission at 480 nm upon 350 nm excitation. SEM shows that the typical nanowire width amounts to 80–400 nm for >5 µm-long nanowires before (Supplementary Fig. 7a, c) and after (Fig. 3j, Supplementary Fig. 7b, d) UV induced crosslinking, by means of an OSRAM UV-Vis lamp ($\lambda$_max_ 366 nm, 250 W) for 12 h under UHV. Inspection of SEM data sets reveal that nanowires are needle-like, become thinner at the tip, and hang away from the BN substrate. These observations evidence surface-templated nanowires, tentatively promoted by island nucleation in Fig. 3c^47^.

### Photo-crosslinking and thermal conversion into nanographene wires

**Fig. 4:**
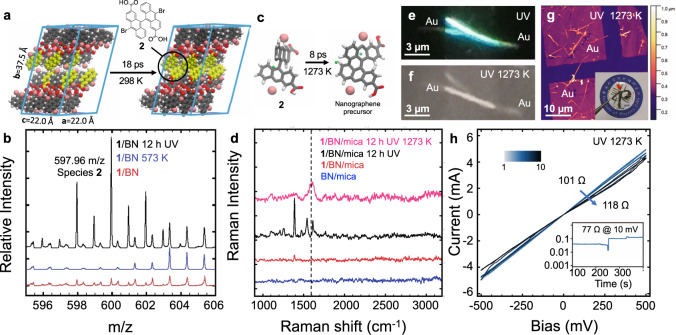
Photo-crosslinking and annealing into nanographene wires. **a** DFTB-MD simulated C–C bond formation at 298 K after debromination. **b** Matrix-assisted laser desorption ionized mass spectrum (MALDI-MS) of **1** after ultraviolet (UV) irradiation on boron nitride (BN) on mica shows formation of species **2** (*m/z* = 597.96). Species **2** is not detected when the sample is annealed at 573 K under argon without UV treatment. **c** The C–H bond breaking DFTB-MD simulation during the high temperature transformation of the supercell in **a** followed by C–C bond formation. Dissociating H atoms are shown in green. **d** Raman spectroscopy of an individual nanowire. The 1389 cm^−1^ anthracene signal intensifies after UV irradiation. After 1273 K 30 min annealing, the 1541 cm^−1^ signal which appears after UV irradiation weakens, and the 1602 cm^−1^ peak (black dash line) broadens. *λ*_ex_ = 532 nm and 2 mW, 60 s integration time. **e**, **f** Photoluminescence (PL) microscopy (480 nm emission at 350 nm excitation) of the nanowire between two gold electrodes after photo-crosslinking, and after 1273 K 30 min annealing. **g** Laser confocal microscopy bright field image of a nanowire. The inset shows a photo of **1**/BN/mica sample with Au electrodes. **h** High current I-V characteristics of a single ~200 nm-wide nanowire reveals a resistance of 101 Ω which increases in time. The inset shows the junction stability at 10 mV and low currents.

Further observations were conducted to address the supramolecular nanowire photo-crosslinking, with key results depicted in Fig. 4. Ex-situ MALDI-MS measurements provide evidence for dimerization of **1** into species **2** after UV irradiation whereby the MS main isotopic distribution is identified as crosslinked species **2**, C_30_H_16_Br_2_O_4_ (calc. *m/z* = 597.94, exp. *m/z* = 597.96, Fig. 4b). Simultaneously, MS signals of the tetrabrominated species C_30_H_16_Br_4_O_4_ (calc. *m/z* = 755.78) are absent, which exclude supramolecular complexes or cycloaddition between the anthracene cores prior to debromination. To rationalize the debromination and dimerization coupling process into species **2**, we perform DFTB-MD simulations at 298 K by artificially removing Br atoms between (**1**)_2_–(**1**)_2_ interlayers. This approximation considers that C-Br photodissociation begins to occur at ~0.1 Å from the equilibrium distance^24^, so that the crystalline structure is not significantly altered when Br (or e.g., Br_2_ or HBr) dissociates and desorbs through vacancies in the molecular architecture during the 12 h of UV treatment under UHV conditions. In the simulations, C–C coupling is observed for interlayers with a twist angle of 90° (Fig. 4a). We note that C–C coupling for different twist angles is possible, but was not investigated in detail due to the related increase of the simulation supercell size. Importantly, polarized Raman (for *λ*_0_ = 532 nm) of UV irradiated structures shows a high degree of anisotropy, pointing towards a topochemical effect of UV irradiation (Fig. 4d, Supplementary Fig. 9a, b, black line). The anthracene peak near 1400 cm^−1^ sharpens after irradiation, contrary to its absence in anthracene photocycloaddition experiments^48^.

DFTB-MD depicts a tentative mechanism whereby heating of the unit cell in Fig. 4a to 1273 K affords C–H bond breaking (Fig. 4c), and nanographene formation (Supplementary Fig. 6). Interestingly, we find that annealing supramolecular nanowires to 1273 K for 30 min is possible by fixing the nanowires between gold electrodes (Fig. 4e–g, Supplementary Figs. 12 and 13). It is worth mentioning that the nanowire will evaporate if annealed without UV treatment (Supplementary Fig. 14). Raman spectroscopy of the UV irradiated **1** on BN/mica annealed 30 min to 1273 K reveals a 70 cm^−1^ FWHM signal centered at 1602 cm^−1^ for *λ*_0_ = 532 nm excitation (Fig. 4d magenta) with a side peak at ~1589 cm^−1^ close to the graphene G peak E_2g_ mode^49^ at *λ*_0_ = 532 nm ~1580 cm^−1^. A G peak FWHM < 100 cm^−1^ together with a notably weak signal near the D peak^50^ (~1410 cm^−1^, FWHM = 60 cm^−1^, D/G intensity ratio < 0.3) is contraindicative of amorphous carbon or high sp^3^ carbon content^51^ at *λ*_0_ = 532 nm. Instead, the weak G peak dispersion (1610 cm^−1^ at *λ*_0_ = 325 nm, Supplementary Fig. 11c) and background shape around the 2D and 2G peaks, supports strained, randomly stacked, or n-doped graphene possibly connected or intercalated by oxygen or bromine atoms^51,52^. For example, absence of 2D peaks and a signal at 1586 cm^−1^ accompanied by a broad 1500 cm^−1^ background has been reported^53^ for a carboxylated nanographene^54^ with 60 sp^2^ carbons. We conclude that the nanowires consist of crosslinked, distinctly-shaped pieces of nanographenes, departing from dehydrogenated species **2** observed in simulations (Fig. 4c). To illustrate this, we summarize in Supplementary Fig. 10 the evolution of the broad 1620 cm^−1^ signal in the powder of **1** and upon self-assembly and UV treatment, from a sharp carbon sp^2^ signal at 1610 cm^−1^ indicative of a highly regular crosslinked material, to a broad signal below 1600 cm^−1^ after annealing.

After 1273 K annealing, PL microscopy reveals PL intensity quenching of the nanowire (close to 480 nm at 350 nm excitation Fig. 4e, f), in agreement with the PL spectroscopy (Supplementary Fig. 15) and a controlled dehydrogenation^33^. Electrical characterization of nanowires at room temperature and under argon flow reveal resistances of (118 ± 44) Ω for electrode-electrode distances of ca. 6 µm, which correspond to a conductivity of (1.6 ± 2.0) × 10^6^ S m^−1^ (19.7 ± 7.3 Ω per μm for a 200 nm diameter nanowire, Fig. 4h), given an approximated cylindrical nanowire diameter of (200 ± 120) nm. We note that slight deviations from a linear ohmic behavior have been observed at very low (~nA) and high currents (~mA), which is attributed to initial Joule heating effects and fatigue at high current densities, respectively. The attained conductivity value of our nanowires is found to be at the order of magnitude of some metal alloys (Galinstan, Constantan) and it is therefore higher than that of CVD or electro-spun carbon nanofibers (10 S m^−1^ to 3 × 10^2 ^S m^−1^, or equivalently 1.1 × 10^5^ Ω to 2.6 × 10^6^ Ω per μm for 200 nm diameter carbon nanofibers)^55,56^ and highly ordered graphene fibers or nanotube bundles (~10^5 ^S m^−1^, 3.1 × 10^−2^ Ω per μm length, for 20 μm diameter graphene fibers)^16,57^. The conductivity of the reported nanographene wires is close to that of graphene formed on the etched sidewalls of silicon carbide, which can achieve ballistic transport up to 10 microns length (i.e., 300 nm width ballistic transport graphene ribbons have a conductivity of ~2 × 10^6 ^S m^−1^, ~10^2^ Ω per μm length of ribbon)^58,59^ and approaches the conductivity of Ag nanowires (~4 × 10^7 ^S m^−1^, 0.2 Ω per μm for a 200 nm diameter nanowire)^60^ and nanotube-Cu nanowires^26^. We found that operating nanowires at higher biases than 0.5 V can lead to enhanced thermal annealing effects further increasing the conductivity. Yet, in most cases the nanowires are damaged, with a breakdown current density of (1.6 ± 1.9) × 10^11 ^A m^−2^ (Supplementary Fig. 16). This breakdown current density is placed between carbon nanofibers (~10^10 ^A m^−2^)^26^ and graphene nanoribbons (~10^12 ^A m^−2^)^61^, in agreement with stacked nanographenes. The advantage of record conductivities and currents density measured in bottom-up nanofabricated structures is multifold: Functional properties, (current) annealing effects, transport physics, and circuitry can be tailored by means of atomic dopants in the precursors, multicomponent self-assembly and photolithography.

In summary, we have studied the self-assembly, photo-crosslinking, thermal conversion and conductivity of anthracene derivatives on BN substrates. The supramolecular architecture precursor self-assembles with four hierarchical levels of interplay: carboxylic acid dimer formation, π-stacking, interrow and interlayer *vdW* interactions, affording 80 to 400 nm-wide supramolecular nanowires. Joint experiment and simulations provide evidence for the formation of nanographene upon UV irradiation and thermal conversion between gold electrodes, resulting in covalently bonded, robust nanographene wires with conductivities of (1.6 ± 2.0) × 10^6^ S m^−1^ approaching those of noble metal nanowires. Our work helps bridge the gap between basic research in on-surface self-assembly and integrated carbon nanodevices, promoting the development of bottom-up templated molecular nanomaterials and crosslinkable organic frameworks (XOFs) with top-down atomic crosslinking precision^62^ directly at device interfaces.

## Methods

### Substrate preparation

The BN surface was prepared either in situ by chemical vapor deposition (CVD) of borazine on Cu(111) under UHV following a protocol described^40^ for the scanning tunneling microscopy experiments, or transferred from commercial BN on Cu foil (Graphene Supermarket) to mica (Changchun Taiyuan Company). First, we coated polydimethylsiloxane (PDMS) on BN/Cu, followed by ammonium persulfate solution for 8 h. After that, the copper was completely etched, and the BN on PDMS was picked up with the mica sheet. After cleaning in deionized water and drying for 1 h, we exfoliated the PDMS very slowly. It is important to note that BN on mica substrates were annealed to 673 K for 1 h under air to remove PDMS residues^63^ until no fluorescence was detected from the substrate. Subsequently the substrates were transferred to UHV.

### Sample preparation

The DBA-COOH molecule **1** was sublimated to the substrate under UHV (5 × 10^−9^ mbar) between 433–453 K for 30 min to prepare a monolayer or multilayer of the molecules, and 60 min to prepare the nanowires. The filament of the evaporator was preheated for 15 min. After sublimation, all samples were flash annealed at 352 K before characterization and irradiated by a 250 W OSRAM HWL lamp without its outer bulb (featuring λ_(UV)_ = 254, 266, 303, 313, 335, 366 nm) under UHV for 12 h (10^−9^ mbar). For the last step, Au(111) electrode pads (30–200 nm thickness) were evaporated. We used a SKY Technology DZ-300 thermal evaporator to evaporate the Au electrodes. The electrode was evaporated through a shadow mask under 5 × 10^−7^ mbar. The sample was then annealed to 1273 K via e-beam heating, employing 600 V sample voltage for 30 min maintaining UV irradiation at high vacuum (10^−8^ mbar).

### Scanning probe microscopy

Scanning tunneling microscopy (STM) was carried out using a CreaTec STM operating between 6 K and 20 K under ultra-high vacuum conditions. Molecules were deposited using a quartz container held between 433 K or 453 K while the BN/Cu(111) substrate^40^ and the sample was kept at 293 K followed by annealing steps described in the main text. All STM data was recorded in constant current mode. The STM images in Fig. 3 were processed using the Gwyddion software^64^, and the Z feedback corrected by a factor of 0.77/3.5, where 0.77 Å is the apparent STM height of the dimer on BN/Cu(111) in Fig. 1 (inset in *i*) under similar tunneling parameters, and 3.5 Å is an estimated height of a molecule lying down on BN/Cu(111). Atomic force microscopy experiments were carried out in a Bruker Multimode 8 AFM in tapping mode under ambient conditions.

### Molecular modeling

The MMFF parameter implementation in the program CHARMM c36^34^ was employed for force field molecular dynamic (MD) simulations. For the in vacuo stacking studies, twelve simulations were performed in a cubic 125 nm^3^ cell with 10 molecules at temperatures between 370 K and 400 K. The trajectories’ π-stacking were analyzed by searching for C-Br carbons at a distance of less than 5 Å from each other. This criterion much larger than the π-stacking distance was employed to account for displaced parallel stacking. For the studies on BN, three layers of trans-(**1**)_2_ (576 molecules) were initialized on a BN mimic^39^ in a simulation cell of size a = 127, b = 132, c = 1000. The Langevin thermostat was employed to perform constant temperature dynamics with a friction coefficient of 0.1 ps^−1^ and a 1 fs integration time step in vacuo, and 0.01 ps^−1^ and 2 fs on the substrate. Density-functional based tight-binding (DFTB) calculations were performed with the DFTB+ package^35^. DFTB is an approximate valence-only DFT method that employs localized atomic orbitals as basis functions. Carbon and oxygen were described by one 2s and three 2p functions and hydrogen by a single 1s function as provided by the 3ob-3-1 parameter set^65^. Bromine was parameterized as described in ref. ^65^. The Nosé–Hoover thermostat was employed for the DFTB-MD simulations with a coupling of 3000 cm^−1^ and a 0.5 fs integration time step. Conjugated gradient geometry optimization search of optimized MD structures was performed using self-consistent charge third-order DFTB with either Lennard–Jones or Grimme dispersion approximation (see text). The self-consistency criterion was set to 10^−5^e. The residual force on each atom was typically smaller than 10^−3^ a.u.

### Scanning electron microscopy

The scanning electron microscopy (SEM) images were scanned by a HITACHI S4800 SEM under 1 kV to 2 kV high voltage, 6000× to 8000× magnification, respectively. We place the sample on a conductive tape on the holder, and in the SEM chamber with a base pressure of 10^−7^ mbar. 1 kV was applied when the sample was not irradiated by the UV lamp, which avoids damage by the electron beam. Samples measured by SEM measured were not characterized further to avoid electron-irradiation related changes in the nanowires.

### Raman spectroscopy

The 325 nm Raman spectra were recorded with a Horiba LabRam HR Evolution Raman spectrometer under 0.5 mW 325 nm laser, and the 532 nm Raman with a Renishaw inVia-Reflex Raman spectrometer under 2 mW 532 nm laser. Both Raman spectra were measured employing a 60 s integration time and 100× Olympus objective.

### Photoluminescence microscopy and spectroscopy

The photoluminescence microscopy (PL) data was captured by an Olympus BX53 PL microscope, whose wavelength of irradiation lamp is 350 nm. We employ a 100× Olympus objective to obtain the optical microscope image and PL microscope image of nanowires, and 5× Olympus objective to obtain the larger scale image of sample. The PL spectra were acquired in a homemade UHV chamber to monitor the process of self-assembly of **1**, UV debromination and thermal conversion. The sample was irradiated by a 337 nm LTB MNL100 nitrogen laser operating at ~80 Hz. The signal was collected by a Princeton Instrument HRS-300 spectrometer and photomultiplier (PMT) detector. The HV of PMT was 1000 V, and the integration time was 1000 ms.

### Matrix-assisted laser desorption ionization and time-of-flight mass spectrometry

A thin layer of (2E)-2-methyl-3-[4-(2-methyl-2-propanyl) phenyl]-2-propen-1-ylidene malononitrile (DCTB) was deposited on top of the samples. A 337 nm laser (LTB MNL100) was employed to ionize and desorb the molecules^24^. The matrix-assisted laser desorption ionization time-of-flight (MALDI-ToF) mass spectrometry (MS) experiments were carried out in a Bruker FLEX2 spectrometers under a pressure of 10^−6^ mbar. The MS of **1** was also measured by a Kore Tech electron ionization ToF-MS after deposition onto a BN/mica substrate. Species **1** was evaporated on BN/mica substrate and the sample was set in front of the skimmer of the ToF-MS. The acquisition time was 30 min, the ionization energy 10 eV and the emission current 200 μA. During the measurement, the temperature of sample was 30 °C at a pressure of 4 × 10^−9^ mbar.

### Electrical characterization

An Imina Micro Probe Station and Keithley 4200A-SCS Parameter Analyzer were employed for electrical characterization of the samples. The samples were exposed to air during transfer from UHV and measured under argon unless stated otherwise, and usually driven for one minute at 10 mV to stabilize the conductivity.

## Supplementary information

Supplementary Information

## Data Availability

All data supporting the findings of this work are available within this paper and its Supplementary Information. Raw data are available from the corresponding author upon request or will be made available at http://palmalab.org/publications.html.
