# Supplementary material for: Self-assembly and photoinduced fabrication of conductive nanographene wires on boron nitride
Source: Nat Commun. 2022 Jan 21;13:442. doi: 10.1038/s41467-021-27600-1 (PMC8782843; doi:10.1038/s41467-021-27600-1)
Supplement: Supplementary file 1 — Supplementary Information [file 41467_2021_27600_MOESM1_ESM.pdf]

Supplementary Information

Self-assembly and photoinduced fabrication of conductive nanographene wires on boron nitride

Xiaoxi Zhang[1,2], Fabian Gärisch[3], Zongping Chen[4,5], Yunbin Hu[4,6], Zishu Wang[1,2], Yan Wang[1,7], Liming Xie[8], Jianing Chen[1,2], Juan Li[9], Johannes V. Barth[10], Akimitsu Narita[4], Emil List-Kratochvil[3], Klaus Müllen[4], Carlos-Andres Palma[1,11]*

[1] *Institute of Physics, Chinese Academy of Sciences, 100190 Beijing, P. R. China*

[2] *School of Physics, University of Chinese Academy of Sciences, 100049 Beijing, P. R. China*

[3] *Department of Physics, Department of Chemistry & IRIS Adlershof - Humboldt-Universität zu Berlin, 12489 Berlin, Germany*

[4] *Max Planck Institute for Polymer Research, Ackermannweg 10, 55128 Mainz, Germany*

[5] *State Key Laboratory of Silicon Materials, Zhejiang University, Hangzhou 310027, P. R. China*

[6] *College of Chemistry and Chemical Engineering, Central South University, Changsha, 410083, P. R. China*

[7] *School of Physics, Beijing Institute of Technology, 100081 Beijing, P. R. China*

[8] *National Center for Nanoscience and Technology, 100190 Beijing, P. R. China*

[9] *Advanced Research Institute for Multidisciplinary Science, Beijing Institute of Technology, 100081 Beijing, P. R. China*

[10] *Physik-Department E20, Technische Universität München, D-85748 Garching, Germany*

[11] *Department of Physics & IRIS Adlershof - Humboldt-Universität zu Berlin, 12489 Berlin, Germany*

**palma@iphy.ac.cn*

Table of Contents

Synthesis of 1 ($Br_2C_{14}H_7COOH$) 3

Supplementary Figure 1 3

Supplementary Figure 2 4

Supplementary Figure 3 5

Supplementary Figure 4 6

Supplementary Figure 5 7

Supplementary Figure 6 8

Supplementary Figure 7 9

Supplementary Figure 8 10

Supplementary Figure 9 11

Supplementary Figure 10 12

Supplementary Figure 11 13

Supplementary Figure 12 14

Supplementary Figure 13 15

Supplementary Figure 14 16

Supplementary Figure 15 17

Conductivity calculation 18

Supplementary Figure 16 20

Synthesis of 1 (Br$_2$C$_{14}$H$_7$COOH)

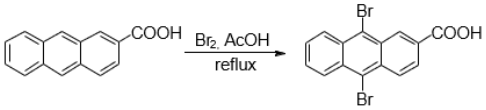

DBA-COOH

Anthracene-2-carboxylic acid (2.01 g, 9.02 mmol) was dissolved in 148 mL of acetic acid at 398 K. Then, bromine (1.9 mL, 37.9 mmol) was added dropwise to the above solution, and the reaction mixture was stirred overnight under reflux. After cooling to room temperature, the resulting precipitates were collected by filtration and recrystallized from THF/MeOH, affording the title compound as a yellow solid (2.67 g, 78% yield). ^{1}H-NMR (300 MHz, THF-d_8, 298K, ppm) δ ^{1}H NMR (300 MHz, THF-d_8) δ 11.87 (br, 1H, COOH), 9.42 – 9.29 (m, 1H), 8.69 – 8.54 (m, 3H), 8.17 (dd, J = 9.2, 1.5Hz, 1H), 7.80 – 7.65 (m, 2H). ^{13}C-NMR (75 MHz, THF-d_8, 298 K, ppm) δ 166.83 (COOH), 132.97, 132.79, 132.20, 132.09, 130.97, 130.77, 129.47, 129.23, 129.05, 128.91, 128.84, 127.45, 125.86, 123.78. High resolution mass spectrometry (HRMS) (TPD, 10eV, 200 μ A, 10^{-9} mbar) m/z: Calc for $C_{15}H_8Br_2O_2$: 377.8891; Found: 377.8853 [M$^+$]. All the reagents and starting materials were commercially available and used without further purification. Nuclear magnetic resonance (NMR) spectra were recorded in THF-d_8 on AVANCE 300 MHz Bruker spectrometers. Thermogravimetric analysis (TGA, Mettler Toledo DSC 3), 10 Kmin^{-1}, 677 K -62.9%; 732 K, -6.7%. HRMS were recorded after **1** evaporated on substrate by a time-of-flight mass spectrometer (Kore Technology), 10eV, 200μA, 10^{-9} mbar.

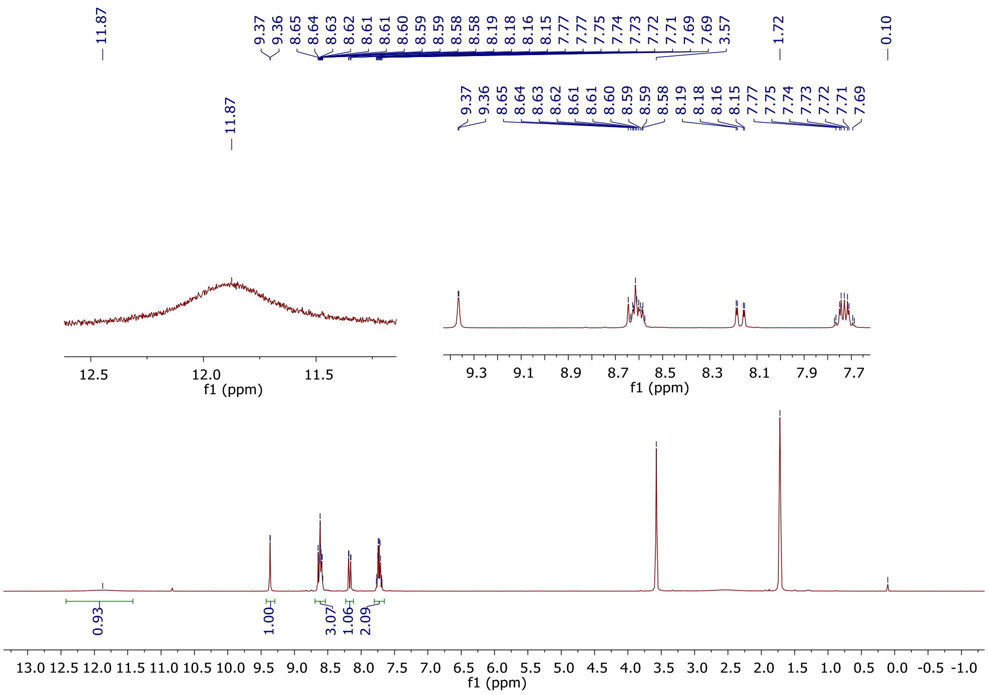

Supplementary Figure 1 ^{1}H-NMR spectrum of DBA-COOH (300 MHz, THF-d_8)

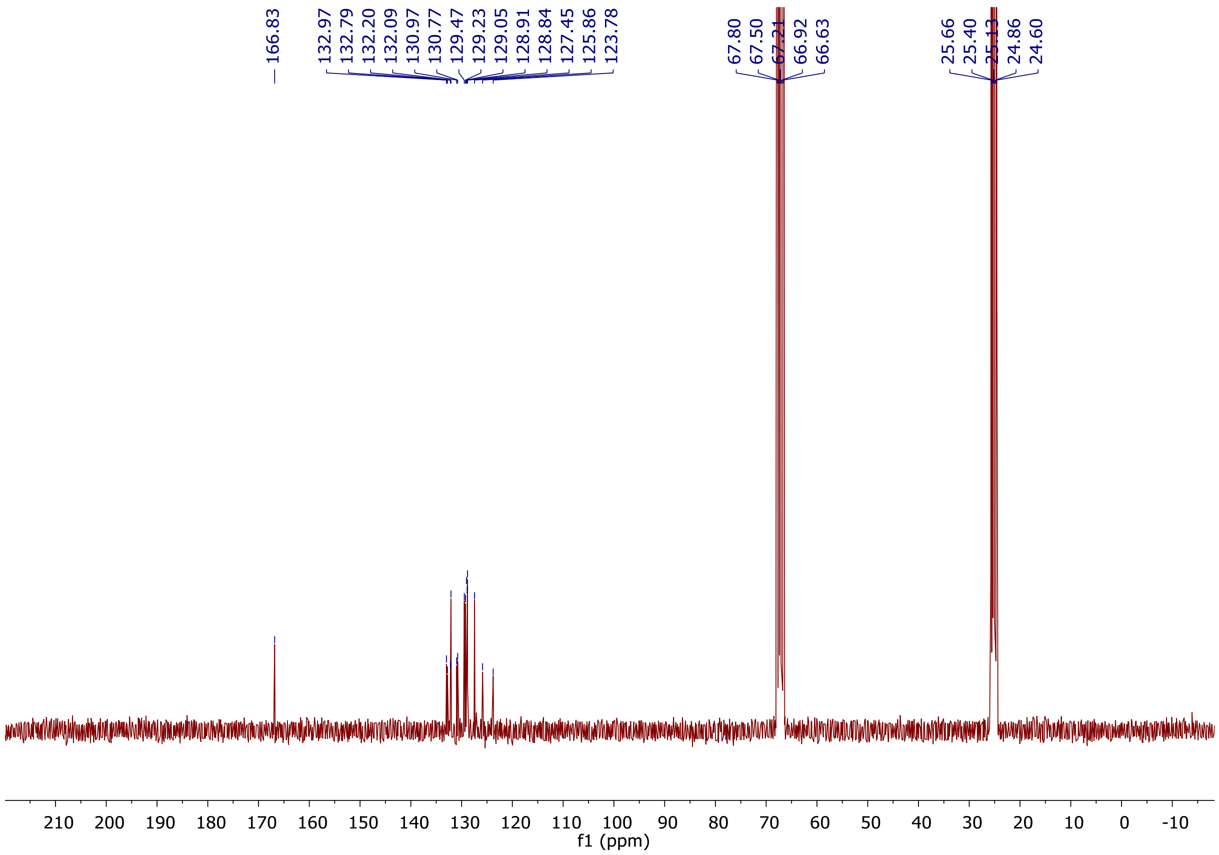

Supplementary Figure 2 ^{13}C-NMR spectrum of DBA-COOH (75 MHz, THF-d_8)

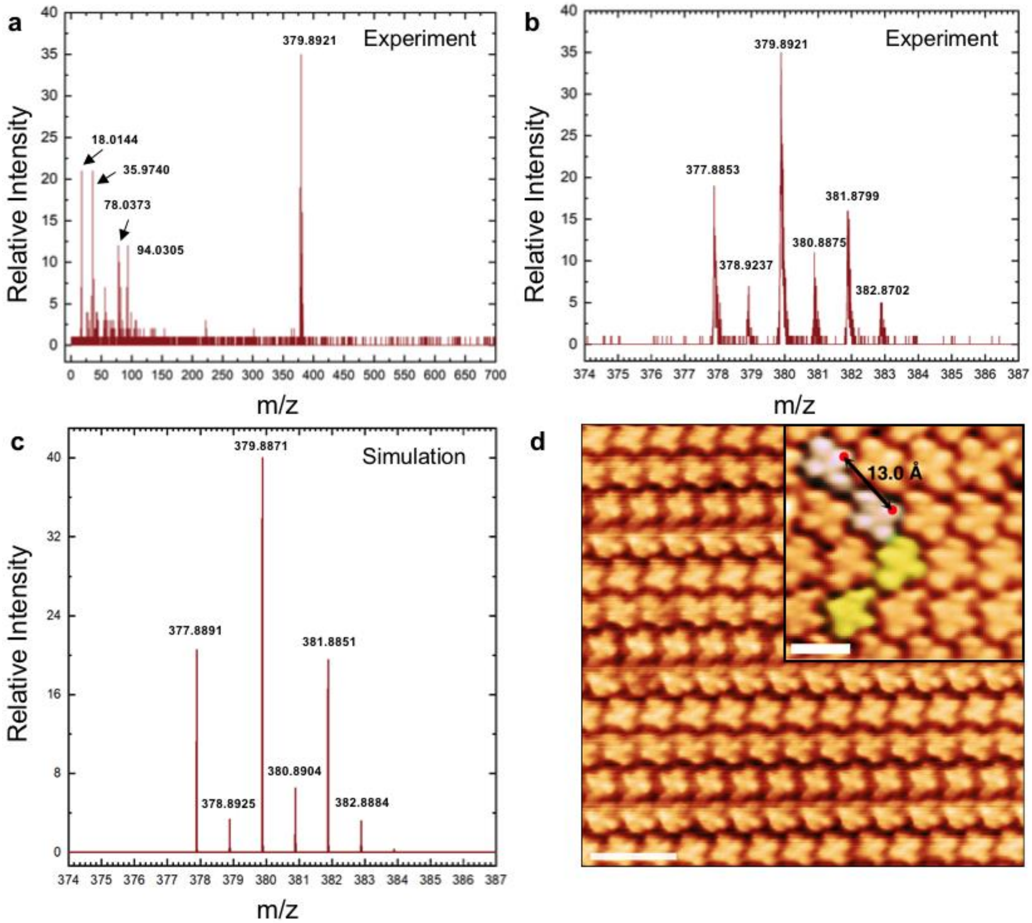

Supplementary Figure 3 The time-of-flight mass spectrometry (ToF-MS) of DBA-COOH monomer and scanning tunnelling microscopy (STM) data for low deposition times. a The full ToF-MS of **1** after the deposition on BN/mica at 453 K measured by thermal desorption electron impact ionization. The peak at m/z = 18.0144 corresponds to H_2O in the UHV chamber. Peaks below m/z = 100 were not identified with fragments of **1** and may correspond to low molecular weight impurities from the chamber. **b** The MS peak of **1** monomer is m/z = 377.8853 (calc. m/z = 377.8891). **c** The simulated MS pattern of **1**. **d** The STM of flat-on DBA molecules on BN/Cu(111) employing low deposition times and a 433 K sublimation temperatures. The STM parameters: I_t = 80 pA, V_s = 1.0 V. Scale bar 2 nm. Inset scale bar 1 nm.

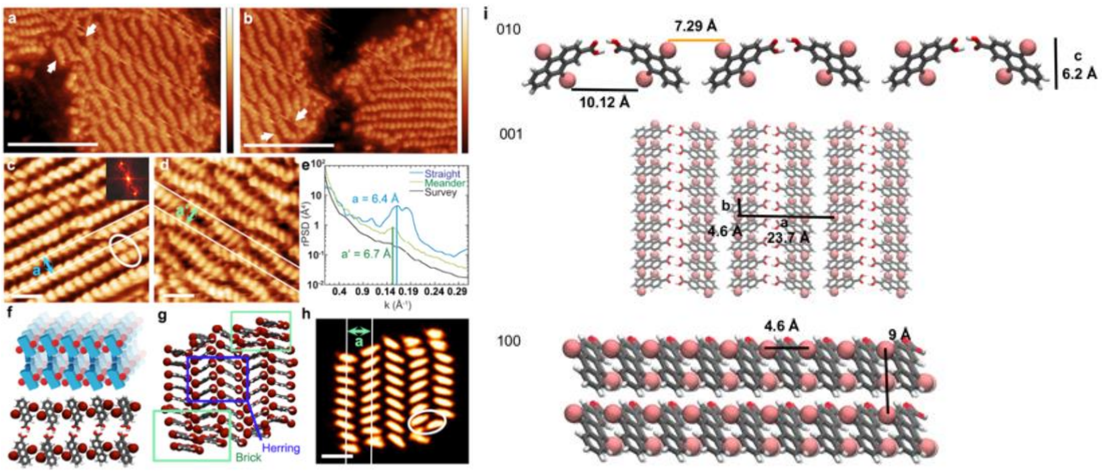

Supplementary Figure 4 Additional scanning tunneling microscopy (STM) data, modelling and alternative self-assembled monolayer. a, b Overview of 20 K ultra-high vacuum (UHV) STM data of islands of **1** after multilayer deposition and mild annealing on BN/Cu(111), whereby white arrows depict a trimeric row and a isolated row. **c, d** STM detail of straight pattern **a** = 6.4 Å and meandering pattern **a** = 6.7 Å (areas marked with green arrows in **a, b**), respectively. **e** Radial power spectral density of data in (**a-d**) reveals a characteristic distance **a**. **f** Side view (with fewer molecules for clarity), **g** top view and **h** molecular orbital plane projection of a 45•trans-(**1**)2 stack optimized via DFTB with **a** = 7.8 Å showing influence of defects by different domain orientations and twisting of single molecule. **i** An alternatively density functional tight binding (DFTB) optimized crystal composed of DBA-COOH dimers cis-(**1**)$_2$ which are not upstanding, resting on their long edge (100), is unable to account for the experimentally observed unit cell vector b nor the apparent single-row patterns in the meandering polymorph. The black line is a guide to the eye. Scale bars: (**a, b**) 5 nm; (**c, d**) 1 nm. STM parameters (**a-d**) I_t = 80 pA, V_s = 0.8 V.

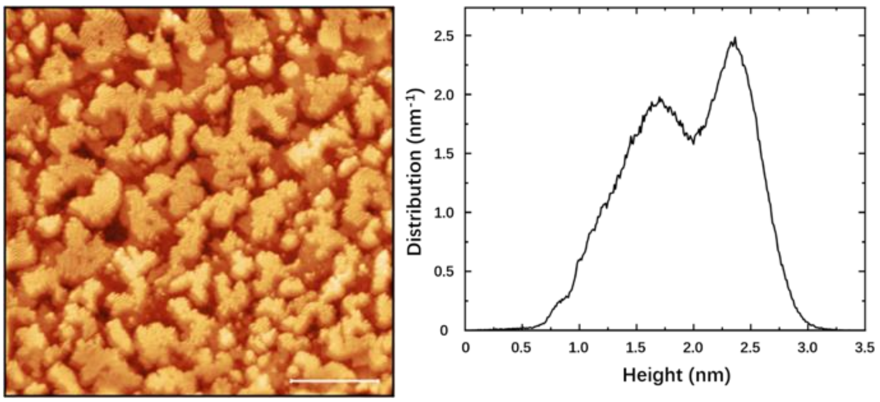

Supplementary Figure 5 Scanning tunnelling microscopy (STM) overview of the multilayer and height distribution. The polynomial background was subtracted from the Raw data without further processing. I_t = 81 pA, V_s = 0.8 V. Scale bar 20 nm.

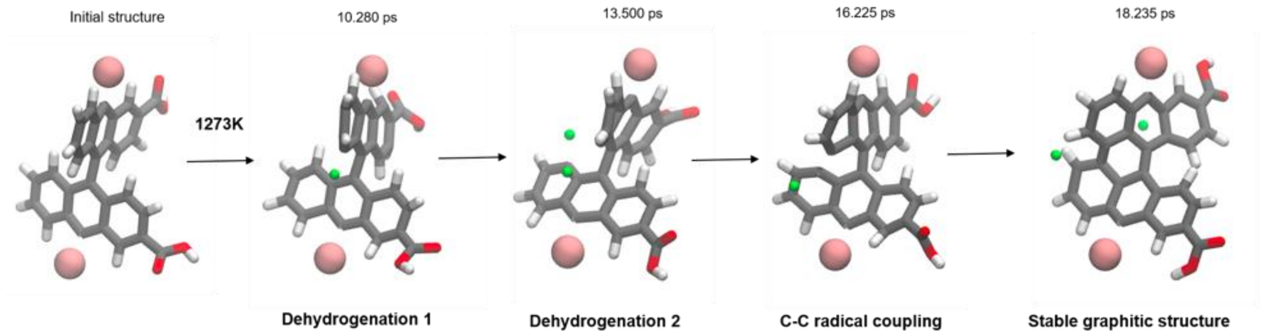

Supplementary Figure 6 Density functional tight binding molecular dynamic (DFTB-MD) at 1273 K. Conversion of Species **2** to nanographene structure was been observed after 18 ps.

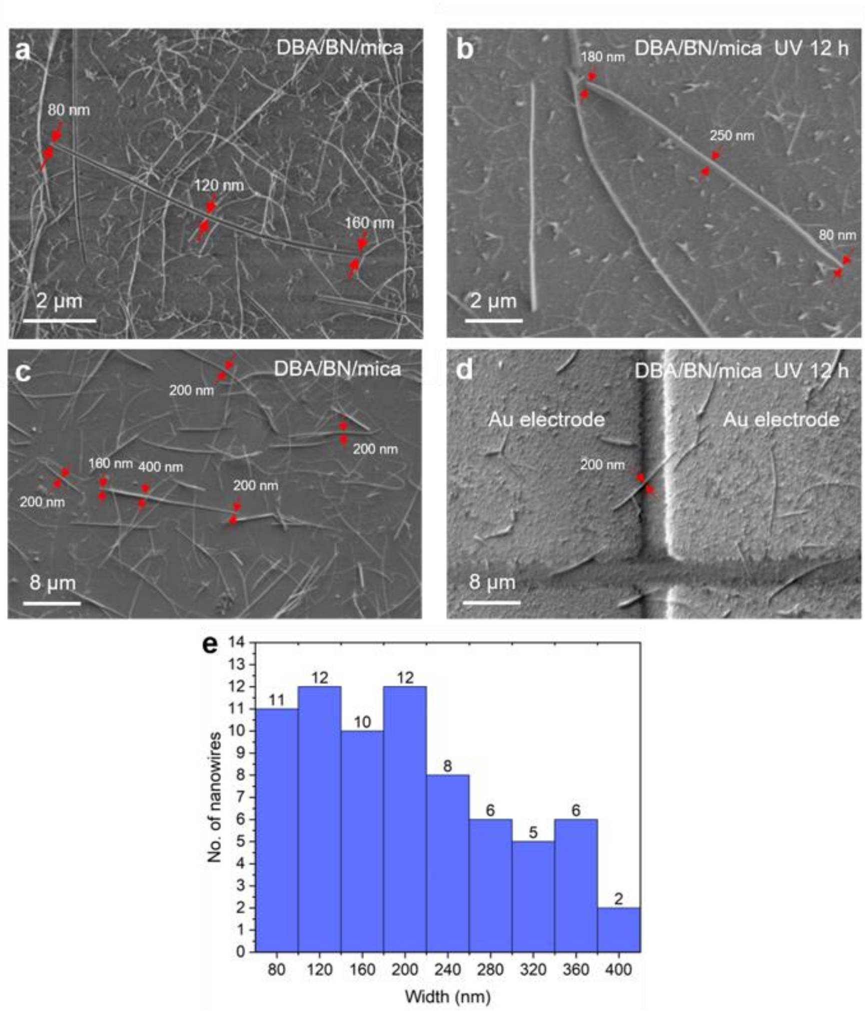

Supplementary Figure 7 The scanning electron microscopy (SEM) of nanowires before and after 12 hours ultraviolet (UV) irradiation. a, c After DBA-COOH molecules sublimation, the substrate is covered by nanowires and thin fibers. SEM HV = 1 kV. **b, d** After 12 hours UV irradiation, the nanowire density is similar while the thin fibers appear to desorb under the influence of the intense 300 W UV radiation, instead of contributing to the ripening of longer or broader crystals. SEM HV = 2 kV. **e** The histogram of number of different widths of nanowires longer than 5 μm from SEM data.

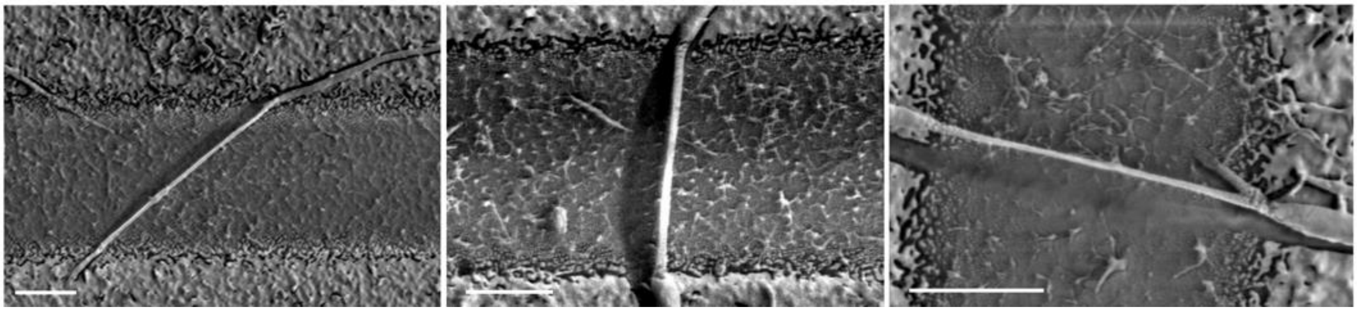

Supplementary Figure 8 The scanning electron microscopy (SEM) of nanowires after 30 min annealing at 1273 K. SEM parameter, HV = 2 kV, I = 10 μA. Scale bars 2 μm,

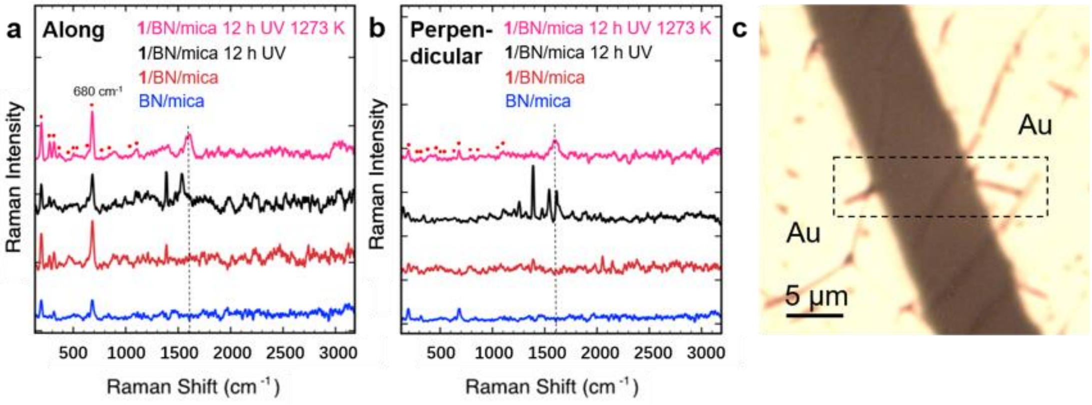

Supplementary Figure 9 Raman of supramolecular nanowire on BN/mica substrate. a, b The polarized Raman along and perpendicular the nanowire. The polarized Raman was excited by the 532 nm 2 mW laser through 100 x objective. And the integration time was standard 60 s. The 1389 cm^{-1} anthracene peak can be observed from all nanowire sample and became stronger after UV irradiation and weaker after 1273 K annealing. The 1542 cm^{-1} peak appeared after UV irradiation both along polarized and perpendicular polarized, but also became weaker after annealing. The G peak centered at 1610 cm^{-1} (black dash line) can be seen in along polarized Raman after annealing, but in perpendicular polarized Raman after annealing, the G peak is at 1602 cm^{-1} (black dash line). Peaks marked by red dots are related to mica. **c** The optical microscope of the nanowire (black dash rectangle) being measured by Raman spectroscopy.

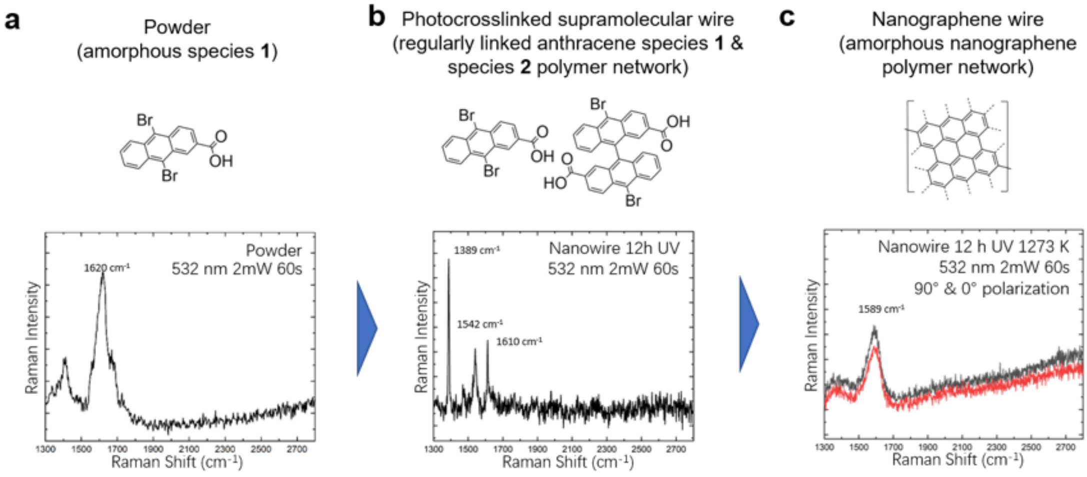

Supplementary Figure 10 Raman of 1 and nanowires after high temperature transformation. a The Raman spectrum of **1** measured on powder showing a broad peak at 1620 cm^{-1}. **b** The Raman spectrum after nanowire self-assembly and ultraviolet (UV) treatment depicts sharp peaks, indicating a highly regular crosslinked anthracene network. **c** After annealing, the G region broadens again, but the D region degreases in comparison to the precursors, indicating successful dehydrogenation and identifying the composition of the nanowire as a nanographene polymer network (see main text discussion). Note that the Raman spectrum in Fig. 4d is a non-polarized Raman spectrum whose G peak is centered at 1602 cm^{-1}, and the Raman in Supplementary Fig. 10c is a polarized Raman spectrum whose G peak is at 1589 cm^{-1}.

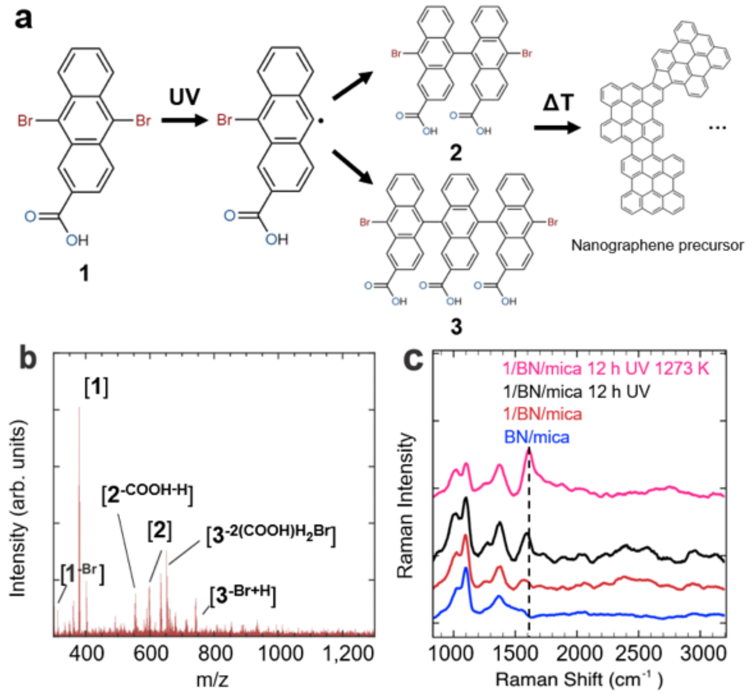

Supplementary Figure 11 Additional mass spectrometry (MS) and Raman. a Schematics of C-Br photodissociation of **1** (m/z = 379.89) and subsequent radical coupling into **2, 3** and a depiction of a nanographene precursors for illustrative purposes. **b** Matrix assisted laser desorption ionization time-of-flight mass spectrometry (MALDI-MS) of **1** and formation of species **2** and **3** on SiO_2. **c** Raman of **1** conducted on BN on mica after sublimation, after 12 h UV irradiation and after UV and 30min-annealing at 1273 K. The peak 1023.43 nm, 1103.86 nm, and 1378.14 nm are from substrate. The 1569.84 nm peak is from **1** and shifts to 1590.53 nm after UV irradiation. After 30 min 1273 K annealing, the peak shifts further to 1610.88 nm (black dash line). λ_0=325 nm, 1 mW.

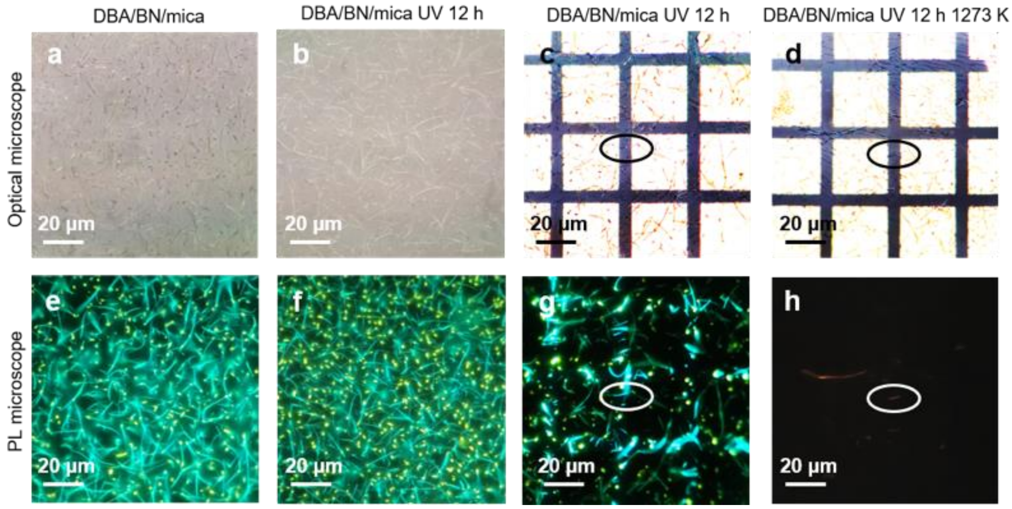

Supplementary Figure 12 The Optical and photoluminescence (PL) microscopy images of nanowire under 12-hour UV irradiation. a, e The microscope of supramolecular nanowires of **1** after sublimation and 353 K flash annealing, the nanowires are everywhere on the BN substrate. **b, f** After 12 hours UV irradiation (254 nm lamp, 300 W), a slight change of color appears (PL peak shift from 480 nm to 484 nm) which can be assigned to few molecules debrominating and photo-crosslinking. **c, g** Evaporation of Au electrodes, showing a nanowire (black and white circle) PL between channels. **d, h** After 30 min 1273 K annealing, few nanowires are left intact (black and white circle).

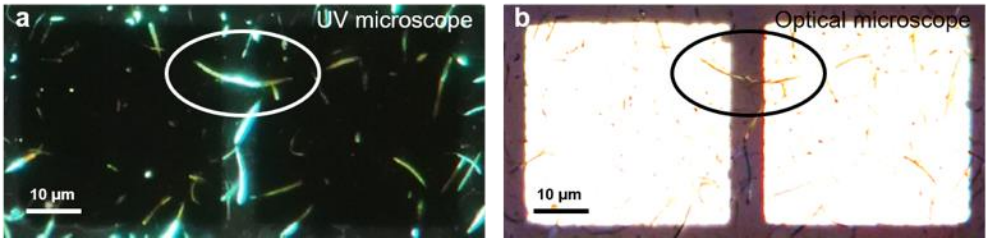

Supplementary Figure 13 Photo-crosslinked nanowires after 573 K annealing. At intermediate annealing temperatures, the ultraviolet (UV) irradiation (12 hours, 254 nm lamp) supramolecular nanowires (black and white circle) enhance their photoluminescence (PL) signal.

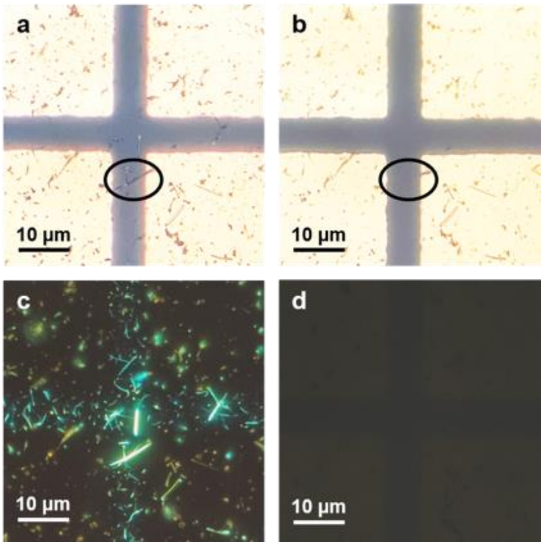

Supplementary Figure 14 The optical microscopy and photoluminescence (PL) microscopy of nanowires before and after annealing without ultraviolet (UV) treatment. a, c The optical and PL microscope before 1273 K annealing. In the black circle there was a nanowire before annealing. **b, d**. The optical and PL microscope of nanowire before and after 1273 K annealing without UV irradiation. Unlike irradiated systems (cf. Supplementary Figure 12), no residual material appears to be left on the surface.

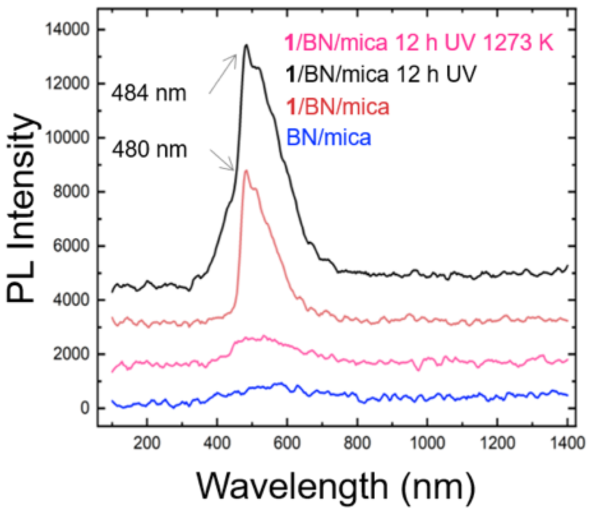

Supplementary Figure 15 Photoluminescence spectrum of 1 on boron nitride (BN) substrate. The BN substrate has a weak but broad photoluminescence and after evaporation of **1**, a PL peak at 480 nm, with 6000 counts, can be observed. After 12 h UV irradiation, the PL peak shifted to 484 nm with the intensity of 8500 counts. Finally, after 1273K annealing, the PL intensity dropped to 1000 counts.

Conductivity calculation

The conductivity was calculated as follows, for a nanographene wire with $R = (118 \pm 44)\ \Omega$, $L = 6 \times 10^{-6}$ m, and $r = (100 \pm 60) \times 10^{-9}$ m.

$$\sigma = \frac{1}{R} \times \frac{L}{\pi r^2} \tag{1.}$$

$$m_\sigma = \sqrt{\left(\frac{\partial \sigma}{\partial R}\right)^2 {m_R}^2 + \left(\frac{\partial \sigma}{\partial r}\right)^2 {m_r}^2} = \sqrt{\left(\frac{-1}{R^2} \times \frac{L}{\pi r^2}\right)^2 {m_R}^2 + \left(\frac{1}{R} \times \frac{-2L}{\pi r^3}\right)^2 {m_r}^2} \tag{2.}$$

Where m_R is the deviation of R and m_r is the deviation of r.

So

$$\sigma = \frac{1}{118\ \Omega} \times \frac{6 \times 10^{-6}\ \mathrm{m}}{\pi(100 \times 10^{-9}\mathrm{m})^2} = 1.62\ \times\ 10^{6}\ \mathrm{S \cdot m^{-1}}$$

Using classical error propagation, the uncertainty is obtained:

$$m_\sigma = \sqrt{\left(\frac{-1}{(118\Omega)^2} \times \frac{6 \times 10^{-6}\ \mathrm{m}}{\pi\left(100 \times 10^{-9}\mathrm{m}\right)^2}\right)^2 \times (44\Omega)^2 + \left(\frac{1}{118\Omega} \times \frac{-2 \times 6 \times 10^{-6}\ \mathrm{m}}{\pi\left(100 \times 10^{-9}\mathrm{m}\right)^3}\right)^2 (60 \times 10^{-9}\mathrm{m})^2}$$
$$= 2.03 \times 10^{6}\mathrm{S \cdot m^{-1}}$$

Which amounts to: $\sigma = (1.6 \pm 2.0) \times 10^6\ \mathrm{S \cdot m^{-1}}$

For the break-down current density, we calculate the new figure with cylinder shape rather than rectangular as follows:

$I_{\text{break down}} = 5 \times 10^{-3}$ A, $r = (100 \pm 60) \times 10^{-9}$ m

$$J_\mathrm{B} = \frac{I_\mathrm{B}}{\pi r^2} \tag{3.}$$

$$m_J = \sqrt{\left(\frac{\mathrm{d}J_\mathrm{B}}{\mathrm{d}r}\right)^2 {m_r}^2} = \sqrt{\left(\frac{-2I_\mathrm{B}}{\pi r^3}\right)^2 {m_r}^2} \tag{4.}$$

Where m_J is the deviation of J_B, and m_r is the deviation of r.

So

$$J_{\mathrm{B}(100)} = \frac{5 \times 10^{-3}\ \mathrm{A}}{\pi(100 \times 10^{-9}\mathrm{m})^2} = 1.59 \times 10^{11}\mathrm{A \cdot m^{-2}}$$

Using classical error propagation, the uncertainty is obtained with:

$$m_J = \sqrt{\left(\frac{-2 \times (5 \times 10^{-3}\mathrm{A})}{\pi(100 \times 10^{-9}\mathrm{m})^3}\right)^2 \times (60 \times 10^{-9}\mathrm{m})^2} = 1.91 \times 10^{11}\,\mathrm{A} \cdot \mathrm{m}^{-2}$$

$J_\mathrm{B} = (1.6 \pm 1.9) \times 10^{11}\ \mathrm{A} \cdot \mathrm{m}^{-2}$.

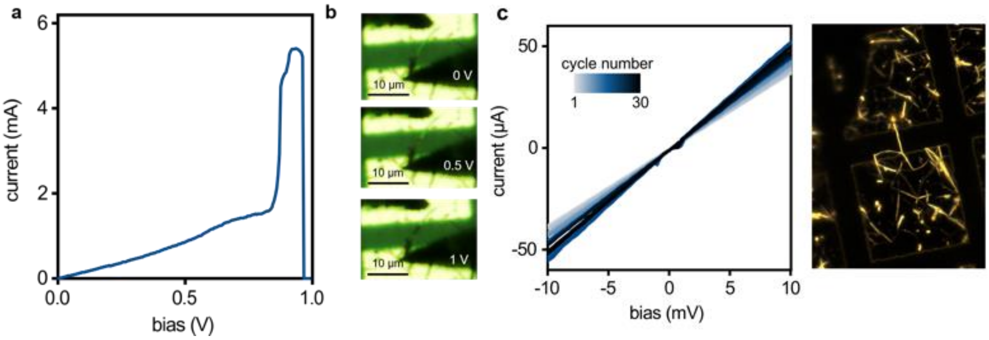

Supplementary Figure 16 I-V curve of nanowire and the process of breaking of a nanowire. a The I-V measurement of nanographene wire shown in Figure 4g. The resistance for this particular nanowire at low bias amounts to 500 Ω after few cycles in air instead of argon, while at higher bias we observe additional thermal annealing followed by a breakdown. **b** The microscope image during the probe station measurement shows partial decomposition (darkening the bottom part of the nanowire) after breakdown to illustrate conductivity through the nanowire. With increasing voltage, the rest of the nanowire darkens until it breaks. No physical contact between probe and nanowire is made. **c** Additional cycles at lower bias for the nanowire depicted in dark field confocal microscopy.
